# Point-of-care ultrasound in prehospital emergency care: attitudes, facilitators and barriers

**DOI:** 10.1186/s12909-026-09500-4

**Published:** 2026-05-26

**Authors:** Alaa O. Oteir, Maram Alakhras, Zainab Alqudah, Maryam Yagbeh, Rasha Buhumaid, Liqaa Rafee, Brett Williams

**Affiliations:** 1 Department of Allied Medical Sciences, Faculty of Applied Medical Sciences, Jordan University of Science and Technology, P.O. Box 3030, Irbid, 22110 Jordan; 2 Department of Emergency Medicine, Mohammed Bin Rashid University of Medicine and Health Sciences, Dubai, United Arab Emirates; 3 Department of Accidents and Emergency Medicine, Jordan University of Science and Technology, Irbid, Jordan; 4 Duke Medical School, National University of Singapore, Singapore, Singapore; 5 Department of Emergency Medical Care, Imam Abdulrahman Bin Faisal University, Dammam, Kingdom of Saudi Arabia

**Keywords:** Point-of-care ultrasound, POCUS, Prehospital, Emergency Medical Services, Emergency Radiology, Barriers, Motivators

## Abstract

**Objective:**

This study aimed to explore Emergency Medical Services (EMS) providers’ perceptions of prehospital Point-of-Care Ultrasound (POCUS), including perceived usefulness, facilitators, and barriers to its implementing in Jordanian prehospital settings.

**Methods:**

A multicenter cross-sectional study was conducted among EMS providers using a structured questionnaire. Attitudes were measured using a four-point Likert scale, while facilitators and barriers were assessed with dichotomous (yes/no) responses. A total attitude score was calculated by summing Likert-scale responses. Descriptive statistics were used to summarize responses and inferential analyses (independent-samples t-tests and ANOVA) were conducted to examine group differences. Internal consistency was evaluated using Cronbach’s alpha and Kuder–Richardson Formula 20 (KR-20).

**Results:**

A total of 621 participants were included in the study. The majority were males (78.3%), with a mean age of 29.5 ± 4.4 years and EMS experience of 9.7 ± 4.6 years. Only 23.3% reported previous POCUS training. Overall, participants demonstrated a net positive attitude toward POCUS (mean score = 27.9 ± 5.4; Cronbach’s α = 0.836), though a substantial proportion (50.0%) expressed uncertainty regarding an absolute POCUS safety claim, a pattern that was independent of prior training status. A proportion of 79.6% expressed willingness to learn about POCUS, 79.4% agreed that POCUS supports standard assessment and 78.8% agreed that POCUS provides additional information for decision-making. The three main reported motivators were training availability (83.1%), direct supervision during use (81.8%) and institutional support (79.4%). The three main barriers were lack of time (76.7%), unavailability of training programs (74.2%) and lack of POCUS experts (73.4%). Participants with prior POCUS training had significantly higher attitude scores (*p* = .001).

**Conclusion:**

EMS providers in Jordan demonstrated a net positive attitude toward POCUS as a potentially useful adjunct to prehospital care, though a substantial proportion expressed safety-related uncertainty, consistent with the absence of routine operational exposure to the technology. Adoption of POCUS remains constrained by structural and training-related barriers. These findings reflect perceived value rather than demonstrated clinical effectiveness. Addressing gaps in training, supervision, competency-based credentialing and imaging governance may support future integration, but further research is required to evaluate clinical and operational outcomes.

## Introduction

Portable diagnostic technologies have been integrated into prehospital care and EMS. Among these portable technologies, POCUS has emerged as one of the most transformative, safe and reliable techniques for EMS providers, enabling real-time assessment of life-threatening conditions such as cardiac arrest, pneumothorax, internal hemorrhage and shock [1–3]. Reviews showed that prehospital ultrasound can enhance diagnostic accuracy and enable prehospital care providers to rule out life-threatening emergencies, expedite triage decisions and support targeted management for trauma, respiratory distress and cardiac arrest [2–4].

Recent studies, from different countries, have demonstrated the feasibility of training prehospital care providers to use ultrasound competently. Studies in the United States, Germany and Canada have shown that, after brief simulation-based training programs, EMS providers have achieved high levels of accuracy in image acquisition and interpretation [5–7].

Despite growing interest in POCUS, robust evidence demonstrating improvements in patient-centred outcomes remains limited. There is a lack of high-quality studies evaluating its impact on key outcomes such as mortality and morbidity [8–10]. The majority of the existing evidence is derived from observational and feasibility studies, with heterogeneous methodologies and inconsistent reporting of clinical outcomes [11]. Although POCUS has been shown to improve diagnostic confidence and occasionally alter management or transport decisions, these benefits have not consistently translated into measurable improvements in patient prognosis [12]. Furthermore, concerns persist regarding potential operational trade-offs associated with prehospital POCUS implementation. These include the risk of workflow disruption, such as interruptions during resuscitation efforts, as well as uncertainty regarding its effects on scene time and overall operational efficiency [8, 9]. Furthermore, challenges continue to exist at the system level. Barriers such as limited governance frameworks, resource constraints and insufficient high-quality evidence further complicate implementation efforts [13].

Importantly, the majority of existing evidence on prehospital POCUS originates from high-income countries with well-established EMS systems, limiting its generalisability to low- and middle-income countries (LMICs). Prehospital care systems in LMICs are often underdeveloped, fragmented and resource-constrained, with variability in provider training, infrastructure and access to equipment [14, 15]. While POCUS has been described as a potentially transformative tool for improving diagnostic capacity in underserved regions, the current evidence base remains limited and heterogeneous, with a predominance of observational studies and a lack of high-quality outcome data [16–18]. Furthermore, implementation in LMIC contexts faces substantial barriers, including limited access to equipment, gaps in training and supervision and the absence of standardized governance and quality assurance frameworks [19–21]. Emerging innovations, such as artificial intelligence–assisted ultrasound, may help address some of these challenges; however, issues related to scalability, infrastructure and integration into existing health systems remain significant [22].

In Jordan, POCUS practical courses were initiated in 2019, offered by emergency physicians and radiologists [23]. However, the routine use of POCUS in prehospital emergency care has not yet been established as a standard practice within the Jordanian EMS system. Prehospital assessment in Jordan continues to rely predominantly on clinical examination and conventional monitoring, with limited integration of real-time imaging at the scene or during transport. This gap persists despite the demonstrated diagnostic and triage advantages of prehospital POCUS in other settings.

To our knowledge, this is the first large-scale, EMS-focused national study in Jordan and the MENA region to explore perceptions of POCUS’s usefulness. Therefore, the current study aimed to explore Jordanian EMS providers’ perceptions towards the usefulness and acceptance of prehospital POCUS. The secondary aim was to assess the key perceived facilitators and barriers influencing its potential implementation.

## Methods

### Study design and setting

This cross-sectional study was conducted between March and June 2024, among EMS providers working at civil defence ambulance stations across various governorates in Jordan. The study followed the Strengthening the Reporting of Observational Studies in Epidemiology (STROBE) guidelines for cross-sectional research.

Prehospital EMS in Jordan is delivered exclusively through the Jordan Civil Defense, which provides a nationally coordinated ground-based response for medical and trauma emergencies across urban and rural areas. EMS providers include paramedics and emergency medical technicians (EMT-B and EMT-I) operating within a protocol-driven system. Currently, ultrasound use is primarily conducted in hospital-based emergency and radiology settings, with no structured prehospital training, credentialing, or governance framework in place.

### Sampling and participants

Using convenience sampling, approximately 800 active EMS personnel were invited to participate in the study. A convenience sampling approach was used due to operational constraints within EMS services, including shift-based staffing and geographic dispersion of ambulance stations. Participants were recruited from multiple stations across different governorates to enhance representativeness.

A minimum sample size of 385 participants was required, assuming a conservative expected proportion of 50%, with a 95% confidence interval and a 5% margin of error [24]. Other evidence recommended a sample of 500 participants for cross-sectional studies [25]. However, these thresholds were expanded to account for incomplete responses and to enhance statistical precision.

EMS providers were included if they were currently employed by the Jordanian Civil Defense and consented to participate in the study. Participants were excluded if they were students, administrative staff, or had incomplete questionnaires.

### Data collection procedure

Data were collected using a structured, self-administered questionnaire. Research assistants visited various civil defense stations across Jordan’s main cities, including Irbid, Amman, Salt and Mafraq. Participants were informed about the purpose of the study, confidentiality of responses and the voluntary nature of participation.

### The questionnaire

The researchers of the current study developed a questionnaire based on previously published literature [23, 26–28]. The questionnaire was reviewed and approved by a group of professionals, including emergency physicians, radiologists, paramedics and radiographers. The questionnaire includes four sections as follows:

A. Demographic and professional characteristics: demographics included age, sex, education level, years of experience and previous POCUS training.
B. Attitudes towards POCUS: usefulness of POCUS section included 10 items on a 4-point Likert scale, with responses ranging from 1 = strongly disagree to 4 = strongly agree. The included items were designed to assess perceived usefulness, value, and clinical relevance of POCUS rather than actual clinical competence or utilization. A total attitude score was calculated by summing responses across all Likert-scale items, with higher scores indicating more positive attitudes toward POCUS.
C. Facilitators of POCUS use: 14 items on a dichotomous scale (Yes/No) assessed institutional, operational, and personal factors that may support the adoption of POCUS in prehospital care.
D. Barriers to POCUS integration: barriers were assessed using nine items with a dichotomous scale capturing challenges related to training, resources, legal concerns and skill maintenance.

### Statistical analysis

Descriptive statistics were used to summarize demographic characteristics and item-level responses, presented as frequencies and percentages for categorical variables and means with standard deviations for continuous variables. Differences in total attitude scores between trained and untrained participants were assessed using independent samples t-tests. Internal consistency for the usefulness scales was evaluated using Cronbach’s alpha. On the other hand, because the facilitator and barrier measures were dichotomized, internal consistency was measured using the KR-20 formula [29]. Data for this study were analyzed by the investigators using the Statistical Package for the Social Sciences (SPSS) version 23.

### Ethical approvals

The study was approved by Jordan University of Science and Technology Institutional Review Board (project number: 20230294).

## Results

A total of 650 responses were received (response rate: 81.3%). Of these, 29 questionnaires were excluded due to substantial missing data (> 80%), resulting in a final sample of 621 participants. Table 1 summarizes the participants’ characteristics. The majority were male (*N* = 486, 78.3%) and the mean age was 29.5 ± 4.4 years. Participants had an average of 9.7 ± 4.6 years of EMS experience, with only 23.3% reporting prior POCUS training (*N* = 145).

**Table 1:** Participants Demographics

Variable	Category	*N* (%)
Sex	Male	486 (78.3%)
	Female	135 (21.7%)
Age (mean (years), SD)		29.5 ± 4.4
Years of experience (year, SD)		9.7 ± 4.7
Educational qualification	High School	106 (17.1%)
	Diploma	447 (72.0%)
	Bachelor’s degree	68 (11.0%)
Previous POCUS training	Yes	145 (23.3%)
	No	476 (76.7%)

*POCUS* Point-of-care ultrasound

### POCUS attitudes

Table 2 includes the responses to the attitude questions. Most participants agreed that POCUS is beneficial for diagnosing internal bleeding (76.5%) and Intravenous (IV) access (72.0%). Agreement on clinical indications was strongly endorsed, including the diagnosis of tension pneumothorax (74.2%) and assessment of cardiac activity (62.9%). Additionally, more than half (78.7%) agreed that POCUS helps enhance clinical decision-making, and 79.4% of participants reported that POCUS supports standard assessment procedures. The mean attitude score was 27.9 ± 5.4, with significantly higher scores among participants with prior POCUS training compared to those without training (29.21 ± 5.0 vs. 27.55 ± 5.5, *p* = .001). The scale demonstrated strong internal consistency (Cronbach’s α = 0.836; 95% CI: 0.816–0.854), supporting the validity of composite score interpretation as the primary attitudinal indicator.

**Table 2:** Prcieved attitudes towards POCUS

Item	Strongly Disagree (*n*, %)	Disagree (*n*, %)	Agree (*n*, %)	Strongly Agree (*n*, %)
1. POCUS is beneficial for IV access	63 (10.1)	111 (17.9)	258 (41.5)	189 (30.4)
2. POCUS is beneficial for diagnosing internal bleeding	44 (7.1)	102 (16.4)	291 (46.9)	184 (29.6)
3. I am interested in learning more about POCUS	48 (7.7)	79 (12.7)	273 (44.0)	221 (35.6)
4. POCUS helps with the standard assessment procedures	37 (6.0)	91 (14.7)	295 (47.5)	198 (31.9)
5. POCUS increases my confidence in my emergency care plan	39 (6.3)	94 (15.1)	297 (47.8)	191 (30.8)
6. POCUS provides additional information for decision-making	36 (5.8)	96 (15.5)	304 (49.0)	185 (29.8)
7. I believe that POCUS does not harm the patient	53 (8.5)	258 (41.5)	221 (35.6)	89 (14.3)
8. I believe that utilizing POCUS is important in my profession	153 (24.6)	167 (26.9)	208 (33.5)	93 (15.0)
9. POCUS is important to assess cardiac activity	107 (17.2)	123 (19.8)	289 (46.5)	102 (16.4)
10. POCUS is important to diagnose tension pneumothorax	38 (6.1)	122 (19.6)	358 (57.6)	103 (16.6)

P*OCUS* Point-of-care ultrasound, *IV* Intravenous

With respect to individual item responses, 50.0% of participants disagreed with the item ‘I believe that POCUS does not harm the patient’ (Strongly Disagree: 8.5%; Disagree: 41.5%). To examine whether this pattern was associated with prior training, a chi-square test was conducted. The analysis revealed a statistically significant but weak association (χ² = 10.49, df = 3, *p* = .015, Cramér’s V = 0.130, *N* = 621). Trained providers disagreed with the safety item at a higher rate (55.8%) than untrained providers (48.3%), with post-hoc comparisons identifying a significant difference in the Strongly Disagree category only (untrained > trained, *p* < .05). When responses were combined to binary format (Agree vs. Disagree), no statistically significant association was identified (*p* = 0.067).

### Facilitators of POCUS use

**Fig. 1:**
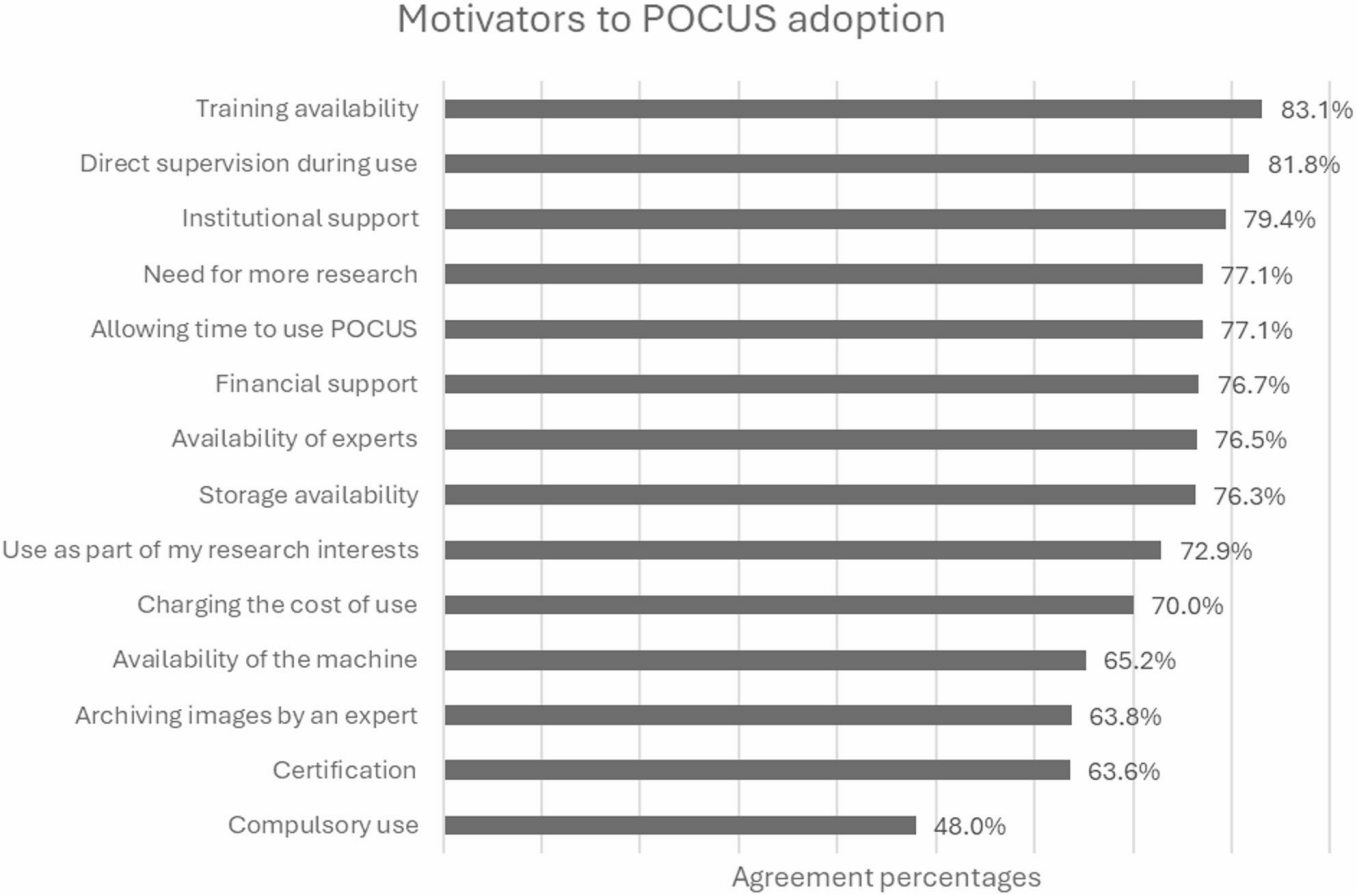
Motivators to use POCUS

Figure 1 outlines perceived facilitators to use POCUS in the prehospital setting. The top five motivators for POCUS adoption were the availability of training (83.1%), direct supervision (81.8%), institutional support (79.4%), the need for research (77.1%) and provision of adequate time to perform POCUS (77.1%). The scale showed high internal consistency (KR-20 = 0.84; 95%CI: 0.821–0.858).

### Barriers to POCUS use

**Fig. 2:**
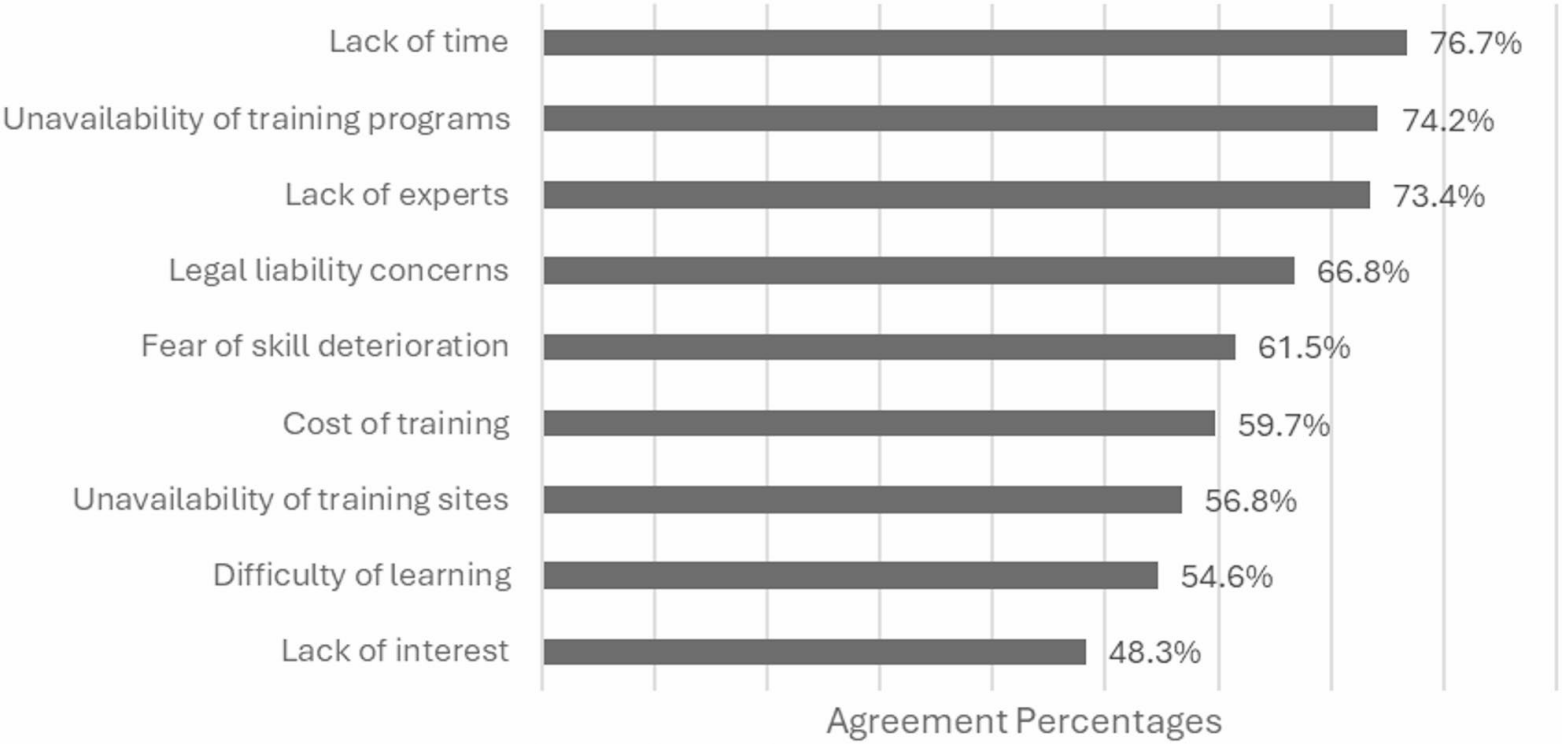
Barriers to POCUS use

As shown in Fig. 2, Key barriers included a lack of time (76.7%), unavailability of training (74.2%) and a lack of experts (73.4%). Legal liability concerns were reported by 66.8%, while fear of skill deterioration was identified by 61.5%. Internal consistency was high (KR-20 = 0.837; 95%CI: 0.818.- 856).

### Previous POCUS training

Participants with prior POCUS training demonstrated significantly more positive attitudes compared to those without training (29.21 ± 4.96 vs. 27.55 ± 5.51, *p* = .001). Trained individuals also reported more facilitators (10.71 ± 2.39 vs. 9.93 ± 3.76, *p* = .003) and fewer barriers (4.72 ± 2.71 vs. 6.03 ± 2.76, *p* < .001) compared to untrained participants. Furthermore, participants without prior training were slightly older than those with training (29.74 ± 4.54 vs. 28.79 ± 4.05 years, *p* = .016). However, no statistically significant differences were observed in years of experience (*p* = .054) or educational level (F(2, 618) = 0.41, *p* = .664) between trained and untrained participants (Table 3).

**Table 3:** Comparison between trained and untrained EMS personnel

Variable (Mean (SD))	Untrained (*n* = 476)	Trained (*n* = 145)	*p*-value
Total Attitude Score	27.55 (5.51)	29.21 (4.96)	0.001
Age (years)	29.74 (4.54)	28.79 (4.05)	0.016
Years of Experience (years)	9.90 (4.71)	9.13 (3.98)	0.054
Facilitators count	9.93 (3.76)	10.71 (2.39)	0.003
Barriers count	6.03 (2.76)	4.72 (2.71)	< 0.001

## Discussion

This study offers one of the first comprehensive national insights into EMS providers’ perceptions of POCUS in Jordan. It identifies both the perceived usefulness of POCUS and the primary facilitators and barriers influencing its potential implementation in the Jordanian prehospital context. Participants generally viewed POCUS as a valuable tool for clinical assessment and decision-making. However, these favorable perceptions may reflect perceived familiarity rather than objective competency, highlighting the need for structured and standardized training programs. Additionally, system-level barriers such as time constraints, limited access to training, and shortages of expert supervision likely indicate structural limitations rather than a lack of clinician acceptance.

An important finding of this study is the significant difference between EMS providers with and without prior POCUS training. Participants who had received training demonstrated more positive attitudes toward POCUS, reported higher perceived facilitators and fewer perceived barriers. This suggests that exposure to ultrasound training and use may enhance perceived usefulness and reduce perceived barriers to its adoption. These findings are consistent with implementation research, where familiarity and training are key drivers of acceptance and perceived feasibility of new technologies. However, these differences likely reflect perceived familiarity rather than objective competence, as this study did not assess clinical performance. Therefore, in a setting where prehospital POCUS is not yet standard practice, these findings suggest that future implementation efforts must prioritize coordinated training, supervision models and imaging governance frameworks.

### Attitudes towards POCUS

A large proportion of Jordanian EMS providers reported positive attitudes regarding the usefulness of POCUS, and those with training demonstrated more favorable attitudes than their untrained counterparts. They supported its potential to enhance diagnostic accuracy, clinical decision-making and early patient assessment. These findings are consistent with previous studies, which reported favorable attitudes among prehospital providers when POCUS’s clinical benefits are well understood [19, 30–32].

Paramedics in various studies demonstrated a strong willingness to adopt ultrasound once proper equipment and training were accessible, confirming POCUS’s role in early decision support during trauma and cardiac emergencies [19, 32]. Similarly, training interventions in Germany and the U.S. have shown that structured, blended-learning curricula significantly improve both theoretical knowledge and hands-on proficiency, allowing paramedics to achieve competence levels comparable to those of medical professionals [5, 6]. The findings of this study indicate that Jordanian EMS personnel are receptive to POCUS and could be competent if they receive similar competency-based training programs.

A notable item-level finding warrants careful interpretation. 50% of respondents disagreed with the statement ‘I believe that POCUS does not harm the patient,’ a pattern that was statistically independent of prior training status. This finding should not be interpreted as evidence that Jordanian EMS providers believe POCUS is inherently harmful. We speculate that the disagreement with such an item may reflect awareness of POCUS’s operator-dependent safety profile, unwillingness to endorse categorical clinical claims or the absence of direct operational experience with the technology. POCUS is not currently integrated into routine prehospital practice in Jordan, meaning the vast majority of respondents were appraising a technology largely outside their clinical experience. Under these conditions, declining to affirm an absolute safety claim is a reasonable response rather than an indication of negative attitude. Importantly, this item-level uncertainty must be contextualized within the overall attitudinal profile, as observed in the composite attitude score being positive across the full sample, with strong scale reliability, and trained EMS providers demonstrating significantly higher overall scores. The composite score, rather than any single item. Nevertheless, the proportion of providers expressing safety-related uncertainty highlights direct implications for implementation planning, as addressed below.

Although there is a positive attitude towards the use of POCUS in the prehospital setting in Jordan, only approximately one-quarter of participants received formal POCUS training. This finding aligns with a global trend of underexposure among paramedics [33]. The lack of standardized training consistently identified a significant barrier to implementation across EMS systems [21]. Previous studies have similarly highlighted how the lack of continuous education and credentialing frameworks limits skill retention and long-term adoption [34, 35].

The findings from this study also highlight a broader international challenge, specifically the development of sustainable, competency-based training for prehospital providers. Taking into consideration the high and unexpected workloads, limited supervision, and life-threatening emergencies that require rapid decision-making. However, feasibility studies demonstrate that even with limited training, paramedics can acquire diagnostically adequate images for cardiac and thoracic conditions [36, 37].

### Facilitators and barriers

The wide range of perceived facilitators for POCUS use in this study is similar to that reported by other studies. For example, training accessibility, direct supervision, institutional support and professional motivation were key predictors of successful integration [21, 34]. The higher count of perceived facilitators among trained participants further supports the notion that exposure enhances awareness of enabling factors, including workflow integration and perceived clinical utility.

Technological advancements can also facilitate POCUS. The emergence of wireless handheld ultrasound devices and the use of artificial intelligence (AI) for image interpretation and integration with cloud-based image review systems can overcome many of the barriers to POCUS adoption, including those in LMIC settings [4, 22]. Currently, POCUS produces high-quality imaging and is supported by telemedicine technologies that may enable remote supervision, quality control and real-time feedback.

Conversely, untrained participants reported significantly more perceived barriers, suggesting that a lack of exposure may contribute to uncertainty and perceived challenges with POCUS use. The primary barriers identified in this study were limited time, insufficient training, lack of expert supervision and medico-legal uncertainty. These barriers align with those reported in studies from other countries [21]. Furthermore, recent research has emphasized the need for image archiving systems and governance frameworks to ensure quality assurance and continuous feedback [38].

The implementation of POCUS faces several challenges, including limited access to POCUS equipment [34, 39] and the need for training to acquire and retain skills [21, 40]. POCUS is also a user-dependent technique that requires sufficient training and periodic quality assurance [41–43]. These challenges highlight the need to invest in education, infrastructure and governance. These include structured feedback systems, image archiving and continuous professional development [38, 39].

### Technology adoption in EMS

The integration of POCUS occurs within the broader context of digital transformation in EMS systems. The prehospital environment has seen increasing adoption of telemedicine, AI-assisted triage and augmented reality training platforms. These innovations have the potential to improve diagnostic accuracy, streamline decision-making and enhance continuity of care between the prehospital field and hospitals [44].

POCUS may exemplify this transition from analog assessment to digitally augmented field medicine, providing a real-time, visual decision-making tool at the patient’s side. In addition, successful implementation may depend on device availability, integration, interoperability with electronic patient care records and robust medico-legal governance. As emerging technologies become more interconnected, the next phase of prehospital innovation may involve AI-supported ultrasound interpretation, automated archiving and remote quality assurance.

Although EMS providers perceived POCUS as useful and valuable, it has not yet become a standard practice in prehospital care in Jordan. The primary barrier to implementation is system readiness rather than clinician acceptance, particularly the lack of coordinated training supervision and imaging governance. Strategic collaboration between the Jordan Civil Defence, the Ministry of Health and the Royal Medical Services is therefore critical to enable safe, standardized and scalable prehospital POCUS integration.

The adoption of POCUS in prehospital care should be interpreted within a broader framework of healthcare decision-making that prioritizes not only diagnostic accuracy but also patient outcomes, operational efficiency and system-level feasibility. While POCUS has demonstrated potential to enhance diagnostic confidence, its impact on mortality, morbidity and time to definitive care remains uncertain. The introduction of ultrasound into the prehospital workflow may increase scene time and operational complexity, which could offset its diagnostic benefits in certain scenarios. This highlights the importance of carefully selecting indications, defining the scope of practice and evaluating whether the additional time and resources required for POCUS use are justified within specific EMS systems.

### Implications and future research

This study identified several priority directions for advancing prehospital POCUS implementation in Jordan, where POCUS is not yet approved as a routine prehospital practice. First, intervention-based studies are needed to evaluate the effectiveness of structured prehospital POCUS competency training. This includes simulation-based education, supervised clinical integration and competency-based certification, ensuring skill acquisition, diagnostic accuracy and patient-centered outcomes. The most perceived facilitators were training availability, supervision and institutional support. Therefore, future research should focus on comparing educational delivery models and assessing their impact on skill acquisition and retention as well as on clinical confidence over time. Second, longitudinal, implementation-focused studies are warranted to examine how dominant barriers, particularly lack of time, limited access to training programs and shortages of qualified experts, influence sustained real-world POCUS utilization. At the systems level, future research should evaluate phased national implementation strategies based on formal collaboration between the Jordan Civil Defence, the Ministry of Health, the Royal Medical Services and other healthcare-related stakeholders, with joint leadership from paramedicine, emergency radiology and emergency medicine to standardize the scope of practice, credentialing and quality assurance. Mixed-methods research incorporating utilization metrics, tele-ultrasound, image archiving and qualitative stakeholder perspectives may be essential to guide scalable, safe and legally robust prehospital POCUS adoption across Jordan and the wider MENA region. Importantly, future studies should also evaluate whether the potential benefits of prehospital POCUS outweigh operational trade-offs, including impacts on scene time and workflow.

### Limitations

Several limitations should be considered when interpreting the findings. First, the cross-sectional design precludes establishing causal relationships between attitudes and perceived motivators or barriers. The observed differences represent perceptions at a single point in time and may change with increased exposure to ultrasound training or changes in institutional policies.

Second, the reliance on self-reported data introduces the potential for response bias, including social desirability and recall bias. Participants may have overestimated the positive perception of POCUS usefulness or underreported perceived barriers, potentially affecting the accuracy of the observed estimates. Furthermore, the questionnaire did not include items assessing current POCUS use, access to ultrasound devices or frequency of use. This limits interpretation of confidence-related responses, particularly among participants without prior training and suggests that reported attitudes likely reflect perceived rather than experienced competence.

Third, although the sample size was substantial, the study was conducted in a single national EMS context, which may limit the generalizability of the findings to other emergency care models with different organizational structures, training capabilities, resource availability and regulatory environments. Therefore, extrapolation of these results to international EMS populations should be done with caution. Also, the use of convenience sampling may introduce selection bias and limit representativeness, despite efforts were made to recruit participants from multiple geographic regions.

Finally, the interpretation of the attitude findings in this study should consider that some items used broad terms, which may have been interpreted variably by participants. Moreover, as fewer than a quarter of respondents reported prior POCUS training, responses to items on confidence and clinical use likely reflect perceived rather than experienced competence. Additionally, the item assessing safety perception is negatively framed, requiring respondents to endorse an unqualified absolute safety claim. Disagreement with this item cannot be unambiguously interpreted as belief that POCUS causes harm, as it may equally reflect clinical uncertainty, awareness of operator-dependency as a qualifying condition or unwillingness to endorse categorical claims in a domain where competency-dependent variability is well recognised. Future research should employ multi-item validated instruments distinguishing harm belief, uncertainty, and operator-dependency awareness as separable constructs. This distinction is important, as the study was designed to assess perceived readiness and acceptance rather than actual utilization or performance.

## Conclusion

This study demonstrated that EMS providers in Jordan perceive POCUS as a potentially useful adjunct for prehospital assessment and decision-making, while also identifying perceived training and system-level barriers to its implementation. Although POCUS is not yet established as standard practice in prehospital care in Jordan, the findings indicate that the EMS workforce holds a positive attitude toward POCUS, while also expressing safety-related uncertainty consistent with the absence of routine operational exposure and the operator-dependent nature of the technology. These findings suggest that the primary constraints on adoption are structural and experiential rather than attitudinal. However, these findings report perceived values rather than actual clinical effectiveness. Addressing gaps in training access, supervision, protected time and imaging governance may support safe, standardized and sustainable prehospital POCUS integration. Further research is required to evaluate the impact of prehospital POCUS on patient outcomes, operational efficiency and health system performance before widespread implementation can be recommended.

## Data Availability

The data will be available from the corresponding author on reasonable request.

## Abbreviations

POCUS: Point-of-care ultrasound
EMS: Emergency Medical Services
MENA: Middle East and North Africa
LMIC: Lower and Middle Income Countries
SPSS: Statistical Package for the Social Sciences
STROBE: Strengthening the Reporting of Observational Studies in Epidemiology

## References

[1] Amaral CB, Ralston DC, Becker TK. Prehospital point-of-care ultrasound: a transformative technology. SAGE Open Med. 2020;8:2050312120932706. 32782792 10.1177/2050312120932706 PMC7383635

[2] Moore CL, Copel JA. Point-of-care ultrasonography. N Engl J Med. 2011;364(8):749–57. 21345104 10.1056/NEJMra0909487

[3] Guy A, Bryson A, Wheeler S, McLean N, Kanji HD. A blended prehospital ultrasound curriculum for critical care paramedics. Air Med J. 2019;38(6):426–30. 31843154 10.1016/j.amj.2019.07.013

[4] Hellenthal KE, Porschen C, Wnent J, Lange M. Evolving role of point-of-care ultrasound in prehospital emergency care: a narrative review. Scand J Trauma Resusc Emerg Med. 2025;33(1):126. 40660332 10.1186/s13049-025-01443-x PMC12261549

[5] Jonck C, Weimer AM, Fundel B, Heinz W, Merkel D, Fiedel H, et al. Development and evaluation of a point-of-care ultrasound curriculum for paramedics in Germany–a prospective observational study and comparison. BMC Med Educ. 2024;24(1):811. 39075429 10.1186/s12909-024-05816-1 PMC11285294

[6] Weber A, Misra A, Rodriguez RD, Brotons AA, Mosetti MA, Lewiss RE, et al. Effectiveness of a simulation-based point-of-care ultrasound course for prehospital providers-a single group quasi-experimental study. BMC Med Educ. 2025;25(1):1093. 40691557 10.1186/s12909-025-07675-w PMC12281742

[7] Bhat SR, Johnson DA, Pierog JE, Zaia BE, Williams SR, Gharahbaghian L. Prehospital evaluation of effusion, pneumothorax, and standstill (PEEPS): point-of-care ultrasound in emergency medical services. Western J Emerg Med. 2015;16(4):503. 10.5811/westjem.2015.5.25414 PMC4530907 26265961

[8] Bøtker MT, Jacobsen L, Rudolph SS, Knudsen L. The role of point of care ultrasound in prehospital critical care: a systematic review. Scand J Trauma Resusc Emerg Med. 2018;26(1):51. 29940990 10.1186/s13049-018-0518-x PMC6019293

[9] Mercer CB, Ball M, Cash RE, Rivard MK, Chrzan K, Panchal AR. Ultrasound use in the prehospital setting for trauma: a systematic review. Prehospital Emerg Care. 2021;25(4):566–82. 10.1080/10903127.2020.1811815 32815755

[10] von Foerster N, Radomski MA, Martin-Gill C. Prehospital ultrasound: a narrative review. Prehospital Emerg Care. 2024;28(1):1–13. 10.1080/10903127.2022.2132332 36194192

[11] Warren J, Tamhankar O, Toy J, Schlesinger SA, Liu YT. Use of ultrasound in the prehospital setting: a scoping review. JACEP Open. 2025;6(2):100086. 40103678 10.1016/j.acepjo.2025.100086 PMC11915001

[12] Taheri O, Samain J, Mauny Fdr, Puyraveau M, Desmettre T, Marx T. Contribution of point-of-care ultrasound in the prehospital management of patients with non-trauma acute dyspnea: a systematic review and meta-analysis. Eur J Emerg Med. 2025;32(2):87–99. 39630617 10.1097/MEJ.0000000000001205 PMC11855997

[13] Naeem S, Edmunds C, Hirst T, Williams J, Alzarrad A, Ronaldson J, et al. A national survey of prehospital care services of United Kingdom for use, governance and perception of prehospital point of care ultrasound. POCUS J. 2022;7(2):232. 36896376 10.24908/pocus.v7i2.15739 PMC9983728

[14] Nielsen K, Mock C, Joshipura M, Rubiano AM, Zakariah A, Rivara F. Assessment of the status of prehospital care in 13 low-and middle-income countries. Prehospital Emerg care. 2012;16(3):381–9. 10.3109/10903127.2012.664245 PMC3360803 22490009

[15] Kironji AG, Hodkinson P, De Ramirez SS, Anest T, Wallis L, Razzak J, et al. Identifying barriers for out of hospital emergency care in low and low-middle income countries: a systematic review. BMC Health Serv Res. 2018;18(1):291. 29673360 10.1186/s12913-018-3091-0 PMC5907770

[16] Baloescu C, Parhar A, Liu R, Wanjiku GW. Effect of Point-of-Care Ultrasound on Clinical Outcomes in Low-Resource Settings: A Systematic Review. Ultrasound Med Biol. 2022;48(9):1711–9. 35786524 10.1016/j.ultrasmedbio.2022.04.221

[17] Becker DM, Tafoya CA, Becker SL, Kruger GH, Tafoya MJ, Becker TK. The use of portable ultrasound devices in low-and middle‐income countries: a systematic review of the literature. Tropical Med Int Health. 2016;21(3):294–311. 10.1111/tmi.12657 26683523

[18] Anderson A, Theophanous RG. Point-of-care ultrasound use in austere environments: a scoping review. PLoS ONE. 2024;19(12):e0312017. 39636834 10.1371/journal.pone.0312017 PMC11620461

[19] Alsulami M, Almukhlifi Y, Alsulami A, Al Nufaiei ZF, Alruwaili A, Alanazy A. Implementing prehospital ultrasound at the Saudi red crescent authority: perceived barriers and training needs. J Multidisciplinary Healthc. 2024:2871–8. 10.2147/JMDH.S457429 PMC11180463 38881755

[20] Delaney PG, De Vos S, Eisner ZJ, Friesen J, Hingi M, Mirza UJ, et al. Challenges, opportunities, and priorities for tier-1 emergency medical services (EMS) development in low-and middle-income countries: A modified Delphi-based consensus study among the global prehospital consortium. Injury. 2025;56(1):111522. 38599953 10.1016/j.injury.2024.111522

[21] Ginsburg AS, Liddy Z, Khazaneh PT, May S, Pervaiz F. A survey of barriers and facilitators to ultrasound use in low- and middle-income countries. Sci Rep. 2023;13(1):3322. 36849625 10.1038/s41598-023-30454-w PMC9969046

[22] Kim S, Fischetti C, Guy M, Hsu E, Fox J, Young SD. Artificial Intelligence (AI) Applications for Point of Care Ultrasound (POCUS) in Low-Resource Settings: A Scoping Review. Diagnostics. 2024;14(15):1669. 39125545 10.3390/diagnostics14151669 PMC11312308

[23] Yagbeh M, Oteir AO, Alakhras M, Alqudah Z, Rafee L, Al-Mnayyis A et al. Exploring knowledge, attitude and confidence in point-of-care ultrasound among emergency physicians and radiologists in Jordan: a cross-sectional study. Emerg Radiol. 2025:1–9. 10.1007/s10140-025-02340-7 40254692

[24] Cochran WG. Sampling techniques. Wiley; 1977.

[25] MacCallum RC, Widaman KF, Zhang S, Hong S. Sample size in factor analysis. Psychol Methods. 1999;4(1):84.

[26] Bashir K, Azad AM, Hereiz A, Bashir MT, Masood M, Elmoheen A. Current use, perceived barriers, and learning preference of point of care ultrasound (POCUS) in the emergency medicine in Qatar–A mixed design. Open Access Emerg Med. 2021:177–82. 10.2147/OAEM.S304153 PMC8140913 34040459

[27] Chakraborty A, Ashokka B. A Practical Guide to Point of Care Ultrasound (POCUS): Springer Nature; 2022 2022.

[28] Wong J, Montague S, Wallace P, Negishi K, Liteplo A, Ringrose J, et al. Barriers to learning and using point-of-care ultrasound: a survey of practicing internists in six North American institutions. Ultrasound J. 2020;12(1):19. 32307598 10.1186/s13089-020-00167-6 PMC7167384

[29] Nunnally JC, Bernstein I. Psychometric Theory McGraw-Hill New York. The role of university in the development of entrepreneurial vocations: a Spanish study. 1978.

[30] El Sayed MJ, Zaghrini E. Prehospital emergency ultrasound: a review of current clinical applications, challenges, and future implications. Emerg Med Int. 2013;2013(1):531674. 24171113 10.1155/2013/531674 PMC3792527

[31] Scharonow M, Weilbach C. Prehospital point-of-care emergency ultrasound: a cohort study. Scand J Trauma Resusc Emerg Med. 2018;26(1):49. 29914554 10.1186/s13049-018-0519-9 PMC6006664

[32] Lobo MJCD, Tavares SCCNM, Pereira de Almeida RP. Point of care prehospital ultrasound in Basic Emergency Services in Portugal. Health Sci Rep. 2022;5(5):e847. 36189415 10.1002/hsr2.847 PMC9489087

[33] Bajwa D, Price J, Boutin S, Kapur A. Prehospital Standards for Point of Care Ultrasound: A Brief National Review. Int J Paramedicine. 2024;5:28–33.

[34] Akanuwe J, Siriwardena AN, Bidaut L, Mitchell P, Bird P, Lasserson D, et al. PP33 Use of point of care ultrasound in prehospital care: an interview study. BMJ Publishing Group Ltd and the British Association for Accident. 2022.

[35] Sedlakova A, Olszynski P, Davis P, Froh J. Prehospital ultrasound use among Canadian aeromedical service providers–A cross-sectional survey. Can J Emerg Med. 2020;22(3):338–41. 10.1017/cem.2019.451 31813395

[36] Rooney KP, Lahham S, Lahham S, Anderson CL, Bledsoe B, Sloane B, et al. Pre-hospital assessment with ultrasound in emergencies: implementation in the field. World J Emerg Med. 2016;7(2):117. 27313806 10.5847/wjem.j.1920-8642.2016.02.006 PMC4905867

[37] Schoeneck JH, Coughlin RF, Baloescu C, Cone DC, Liu RB, Kalam S, et al. Paramedic-performed prehospital point-of-care ultrasound for patients with undifferentiated dyspnea: a pilot study. Western J Emerg Med. 2021;22(3):750. 10.5811/westjem.2020.12.49254 PMC8203026 34125056

[38] Naeem S, Aziz S, Hirst T, Strobel J, Mulvey JM, Lang A, et al. Implementation of prehospital point-of-care ultrasound using a novel continuous feedback approach in a UK helicopter emergency medical service. Scand J Trauma Resusc Emerg Med. 2025;33(1):21. 39905531 10.1186/s13049-025-01340-3 PMC11796228

[39] Maw AM, Galvin B, Henri R, Yao M, Exame B, Fleshner M, et al. Stakeholder Perceptions of Point-of-Care Ultrasound Implementation in Resource-Limited Settings. Diagnostics. 2019;9(4):153. 31635219 10.3390/diagnostics9040153 PMC6963438

[40] Arnold MJ, Jonas CE, Carter RE. Point-of-Care Ultrasonography. Am Family Phys. 2020;101(5):275–85. 32109031

[41] Supino MC, Buonsenso D, Scateni S, Scialanga B, Mesturino MA, Bock C, et al. Point-of-care lung ultrasound in infants with bronchiolitis in the pediatric emergency department: a prospective study. Eur J Pediatrics. 2019;178:623–32. 10.1007/s00431-019-03335-6 30747262

[42] Mumoli N, Vitale J, Giorgi-Pierfranceschi M, Sabatini S, Tulino R, Cei M, et al. General practitioner–performed compression ultrasonography for diagnosis of deep vein thrombosis of the leg: a multicenter, prospective cohort study. Annals Family Med. 2017;15(6):535–9. 10.1370/afm.2109 PMC5683865 29133492

[43] Schott CK, LoPresti CM, Boyd JS, Core M, Haro EK, Mader MJ, et al. Retention of point-of-care ultrasound skills among practicing physicians: findings of the VA National POCUS Training Program. Am J Med. 2021;134(3):391–9. e8. 32931765 10.1016/j.amjmed.2020.08.008

[44] Marsh-Feiley G, Eadie L, Wilson P. Paramedic and physician perspectives on the potential use of remotely supported prehospital ultrasound. Rural Remote Health. 2018;18(3):1–19. 10.22605/RRH4574 30207737

